# Are surveys blind to sexual and gender diversity? Reflections and an open proposal

**DOI:** 10.3389/fpsyg.2024.1369214

**Published:** 2024-06-04

**Authors:** Raquel Royo, Iratxe Aristegui, Maria Silvestre

**Affiliations:** Social and Human Sciences Department, University of Deusto, Bilbao, Basque Country, Spain

**Keywords:** intersectionality, gender, sexualities, quantitative methodology, identities, indicators

## Abstract

This article presents an open proposal on how to include questions that capture different gender identities and sexual orientations in quantitative research. Our theoretical framework is feminist theory and the evolution of feminist debates on identity categories, where the introduction of an intersectional gender perspective has been an important paradigm shift. We have compiled different previous categorization proposals and consider the consequences of not including categories that reflect identity diversity in surveys in order to finally offer our proposal for operationalizing identities. The proposal aims to ensure comparability in longitudinal studies and, at the same time, to incorporate new identity frameworks and an intersectional perspective in quantitative methodology research.

## 1 Introduction

This article is based on the following research question: Is it possible to include new sociodemographic questions that capture diverse gender identities whilst maintaining the comparability of longitudinal studies? The initial response to this question was yes, however, another question immediately arose: How can this be done to not only capture the diversity of identities but also the feminist epistemological disparity in this area? Here the response was more complex. We have taken as a starting point the feminist debate on gender categories, sexualities and intersectionality. We consider that the incorporation of an intersectional gender perspective is an important milestone in feminist epistemology which involves taking on pending methodological challenges as, in most cases, the decision to include diversity and break with androcentrism has been made through qualitative methodologies. Nonetheless, quantitative methodology also needs to take on the challenge to measure, explain and consider identity diversity. The article goes on to examine the different ways of operationalizing sex and gender variables in surveys. We also consider the consequences of not including new variables and categories in surveys and for this purpose we focus on the European Values Study. Finally, we present our proposal of categories; a proposal that is open to scrutiny and debate.

## 2 Feminist debates on categories: gender, sexuality and intersectionality

The categories and concepts used in the social sciences and in everyday life are not mere neutral, natural and immutable definitions that reflect reality as if it were a mirror, but social and historical constructs. This implies that such constructs have developed and evolved over time and hold a particular meaning in a specific historical place and time (Royo, 2012).^1^ In the field of gender and sexualities, the last decades have seen the questioning of relevant concepts such as sex, gender or women, and the popularization and proliferation of new categories, for example, intersexual, cisgender, heteronormativity, transgender, transexual, *queer*, non-binary. This has shaken the foundations of gender binarism and has turned the very subject of feminism into the object of debate, shaping one of the most controversial debates in feminist theory and praxis in the last few decades. This section provides a brief contextualization in order to understand the principal categories related to gender and sexualities and the debates around them.

The notion of gender has challenged biological determinism by revealing that feminine and masculine are not natural or biological facts but social constructs (Cobo, 1995). Although the development of gender as a concept is relatively recent, the idea that inequality between women and men does not come from nature has a long and hidden history, and refers to feminist theory (León, 2015). To name a few examples, we can turn to Poulain de la Barre who, as early as the 17th century, considered female inferiority a prejudice “as old as the world” (Poulain de la Barre, 1984, p. 9); to the enlightened Mary Wollstonecraft, who rejected the idea that sexual difference is arbitrary and would not occur “if women were not oppressed from the cradle” (Wollstonecraft, 2005, p. 313); to John Stuart Mill, who, similar to Wollstonecraft, asserted that the alleged “nature of women is an eminently artificial thing” (Mill, 2001, p. 171); and, of course, to the existentialist Simone de Beauvoir, and her paradigmatic maxim:

One is not born, but rather becomes, a woman. No biological, physic or economic destination defines the figure that the human female takes on in society; it is civilization as a whole that elaborates this intermediary product between the male and the eunuch that is called feminine (De Beauvoir, 1972, p. 13).

But one would have to wait for the “second wave” of feminism – in the second half of the 20th century – in order for its protagonist, radical feminism, to systematize and diffuse the concept of gender, which Millet understands as “personality structure in terms of sexual category” (Millet, 2000, p. 29).^2^ Thus, the notion of gender, that would later be used in United Nation conventions and in the institutional sphere, links feminist theory to the division of power and to the patriarchy, fundamental aspects of radical feminism (Oliva, 2005). The concept of gender arose in opposition to sex within a framework of binary opposition. It can be defined as a combination of practices, beliefs, representations and social norms that emerged from members of a human group based on the symbolization of the anatomical difference between women and men, whilst sex alludes to the anatomical and physiological characteristics that differentiate the human female from the human male (León, 2015).^3^

Over the last decades, the concept of gender has been the focus of intense debate (Oliva, 2005). Since the 1970s, Black and Chicana feminist voices from the United States (Combahee River Collective, 1981; Hooks, 1981; Moraga and Andalzúa, 1981; Davis, 1983; Andalzúa, 1987; Hill Collins, 1990) have rejected the unambiguous use of the female category and the construction of the female norm “based on the experience of white, heterosexual, middle-class, Christian women” (La Barbera, 2016, p. 108). Awareness of the limitations of using gender as a sole analytical category has led to a broad consensus on the need to adopt an intersectional approach in feminist analysis (Nash, 2010, cited in Gandarias, 2017, p. 74), and intersectionality (Crenshaw, 1989) has become a mark of identity of the third and fourth waves of feminism (Silvestre et al., 2021). “Intersectionality is a method, a disposition, a heuristic and analytical tool” (Carbado et al., 2013, p. 303); a way of understanding and analyzing the complexity of the world, people and human experiences, that allows us to comprehend that social and political events, and one’s own subjectivity, are shaped by many factors or categories in diverse and mutually influencing ways (Hill Collins and Bilge, 2016), and impact an individual’s life and identity in ways that exceed the sum of its parts (Severs et al., 2017). In order to understand the complexity of relationships, social problems, people and the notion of identity itself, Platero proposes the image of a “tangled mess” (2013: 45) that provides us with a “multifaceted gaze.” This perspective allows us to appreciate the intersectional nature of our lived experiences and identify hidden experiences of subordination or privilege in a specific sociohistorical context (Platero, 2012).

Similarly, we can also refer to theoretical developments that, rather than considering sex as something biologically determined and gender as something that is culturally acquired, affirm that both are socially constructed (Giddens, 2002, p. 158).^4^ From this perspective, *sexuality* emerges as a social and historical construction that constitutes a new field of study (Maquieira, 2001, pp. 173, 177). Rubin (1989) is one of the classical theorists that promotes this line of thought and argues that a politics of sexuality independent of a politics of gender is essential.^5^ According to Rubin there is “a hierarchical system of sexual value” (1989, 136) in modern Western societies that hierarchically classifies the following categories: monogamous heterosexual men and women, bound by a monogamous marriage with children; unmarried monogamous heterosexual men and women, with or without offspring, followed by most other heterosexuals; gays and lesbians in stable relationships, promiscuous gays and lesbians and, finally, the most despised sexual castes that includes, among others, transgender people, fetishists, sadomasochists or prostitutes. For Momoitio (2019), the idea that underpins this erotic pyramid is still current and can be summarized as “there are sexual-affective relationships that are more valued than others” (2019: 55),^6^ whilst Platero (2012, p. 18) points out that “sexualities stigmatized as “abject” or “belonging to the margins” or “dissident” (…) are exactly those that help us to understand how power and privilege work in all sexualities and in all individuals.”

Theorists such as Rivera Garretas (1994, p. 168) have highlighted that the concept of gender – and specifically the emphasis on the relational dimension of femininity and masculinity – is primarily rooted in heterosexual sexuality. “Lesbian feminism emerges expressly as a challenge to the gender category, as a critique to the essentialist definitions that speak of women from a heterosexual experience”^7^ (Álvarez, 2001, p. 275) and it rejects compulsory heterosexuality as a political institution (Rich, 1999; Wittig, 2009). Rich (1999) theorizes “the lesbian existence” in history and “the lesbian continuum,” a host of experiences that shape a sense of feminine homosexuality throughout a woman’s life (Rich, 1999), while Wittig (2009) affirms that the heterosexual norm oppresses by rejecting all discourse that escapes that logic. She sustains that “lesbians are not women,” as women “only have meaning in heterosexual systems of thought and heterosexual economic systems” (Wittig, 2009, p. 143), thus advocating for “a political transformation of concepts” (Wittig, 2009, p. 141).^8^

Within the framework of radical anti-essentialism, queer theory proposes a complete break from identity categories and questions everything that hegemonic narratives consider valid, true and immutable. This perspective interrogates traditional categories of sex, gender and sexuality and assumes that, among other things, the term *queer* can not only be applied to lesbian women and gay men, but it also encompasses bisexual, transexual and intersexual individuals, even heterosexuals whose practices transgress normative models (Jackson and Scott, 2010, cited in Suarez, 2019).

Butler (1990), well known for their contributions to queer theory, describes gender as a *parody* and defends the inclusion of all discourses on sex (*ironic games*) to destabilize gender. For Butler (2022, p. 15), “performativity must not be understood as a singular or deliberate act, but rather as the reiterative practice by which discourse produces the effects that it names.” For Guerra and Fernández (2022), Butler’s most notorious and polemical idea is precisely the one that states that sex is a social construction, an historic category, so that, in Butler’s words, “the distinction between sex and gender is shattered,” that is, “what, if anything, is left of sex, once it has assumed its cultural character and has become gender?” (Butler, 2022, 19). Gender would emerge as a term that would absorb and displace sex, reducing it to fiction or linguistic fantasy. The regulatory norms of “sex” operate in a performative way to construct the materiality of the sex of the body (sexual difference), “in order to consolidate the heterosexual imperative” (*ibid*, 15). Thus, Butler does not interpret sex as something that one has, nor as an immutable description of what one is, nor as “a bodily given on which the construct of gender is artificially imposed, but as a cultural norm which governs the materialization of bodies” (*ibid*, 15).

The formation of the subject takes place by assuming a sex, that is, by identifying with the normative specter of sex by rejecting “abject beings,” those who are not subjects and constitute the outside of the subject’s world (and inside the subject as its very abhorrence that constitutes it) (*ibid*, 15). Therefore, as Bagiotto (2019) synthesizes, gender is a performative practice that does not start from the nature of sex/body, but it acts on this norm, determining its formation and characteristics. In repetition, gender generates itself as a – heterosexual – norm. By following the heteronormative gender, the sex/body in turn reinforces the norm and thus they are both culturally co-produced; “the norm regulates the body, and the body regulates the norm” (Bagiotto, 2019).^9^

For Coll-Planas (2010), there are three distinct ways of understanding sex and gender: (1) Gender as a product of sex, from biological determinism that claims that gender identity emanates from our sexual characteristics (chromosomes, gonads, hormones…); (2) Sex and gender are two relatively separate elements that distinguish the biological dimension (sex), and behavior and personality traits (gender), understood as a social construct. This assumes that sex is something immutable in an individual and gender is variable and can be culturally modified, which infers that biology and culture are distinct elements;^10^ (3) Sex as a product of gender, which is the position that Coll-Planas takes, aligning with Butler’s contribution. Thus, gender can be understood as “a social product that shapes human beings into men and women, not only in their behavior and subjectivity, but also in the physical dimension,” questioning the pre-social character of sex (Coll-Planas, 2010, p. 69).

Conversely, binary conceptualizations of gender have led to discourses and explanatory frameworks that capture diverse expressions of masculinity and femininity, such as Raewyn Connell (1995), who studied masculinities structured around hegemonic masculinity that occupy the pinnacle of the gender order and dominate all femininities^11^ or Velasco’s (2009) work on traditional and modern femininities and masculinities or those in transition. Marcela Lagarde refers to the “syncretism of gender” (Lagarde y de los Ríos, 2000, p. 45), to describe how contemporary women – and men – are a mixture of traditional and modern gender traits, in a way that this simultaneity of gender cultures, with respect to sexuality or roles “can create contradictory situations, conflicts or subjective paradoxes” (López Sáez and García-Dauder, 2020, p. 22).

As already mentioned, the different theoretical perspectives on gender and sexualities has generated debate between those who, from queer positions, attempt to destabilize these categories that they understand as fluid (Platero, 2012, p. 37) and those who defend “we, the women” as a political subject “necessary to achieve visibility and attainment of rights” (De Miguel, 2014, p. 34).^12^ Similarly, the well-known controversy “recognition or redistribution” that puts at odds the theorists Butler and Fraser (2000), raises discourses “difficult to reconcile,” inasmuch as the former prioritizes the search for solutions to problems of social justice – considering that the politics of redistribution seem to take second place compared with the extension of policies recognizing diversity – whilst the latter defends the centrality of cultural problems, addressing social and political practices (in particular those that affect sexuality) and how these shape new possibilities for life (Galcerán, 2000, p. 8).

Delving deeper into the core of intersectionality, McCall (2005, cited in Platero, 2012), discusses three different ways to approach this perspective that she classifies as: anticategorical, intercategorical and intracategorical. For the first approach, the only way to eliminate discrimination is to abolish the same categories that differentiate and classify people into groups, deconstructing those categories that we consider unquestionable, aligning with the contributions of queer, postcolonial or crip theory. The intercategorical approach attempts to document and analyze inequality within the multiple dimensions and social groups that exist as a consequence of social categories. Finally, the intracategorical approach, which falls between the other two, critiques the usual social categories, without ignoring their importance in understanding society and relationships. The emphasis would be on those individuals who blur the boundaries of these categories to understand the complexity of lived experiences and the social norms that we consider natural (McCall, 2005, cited in Platero, 2012, p. 36).

Nonetheless, “however imposing we want to be with sex and gender categories, they are constantly being rearticulated” (Platero, 2013, p. 48) and this poses new challenges for the social sciences if it does not wish to use obsolete instruments in the face of a changing reality that it seeks to understand.

## 3 How to capture these categories in surveys

As we have just shown, we are faced with a major challenge when thinking about “how” to ask about these questions if we really want to adapt to new realities, and how we can measure them from both a quantitative and qualitative viewpoint.

Official datasets of population diversity in terms of gender are limited and deficient. Statistics are needed not only to capture data on individuals’ sex, gender and sexual orientation but also to bring to the fore new diversities and respond to the United Nations sustainable development objectives of the 2030 agenda to “leave no one behind” (Instituto Nacional de Estadísticas, 2022). Therefore, our aim is to contribute to the development of new questions and response categories that reflect the diversity of bodies, genders and sexual orientation (Stang, 2019).

It is important, therefore, to understand how to formulate survey questions on gender in such a way that all participants feel included and can respond comfortably. It is evident that sexual identity, gender identity and sexual orientation are not the same. Until relatively recently, and still today, a clear conceptual differentiation, and their measurement, are issues that are barely taken into account when developing sociodemographic questions in surveys.

We are interested in advancing and carefully addressing this question as it will allow us to conduct a more exhaustive analysis of respondents and their relationship with specific answers to different topics of study beyond the traditional MALE and FEMALE binary classification of the (biological) sex variable that usually appears in surveys and administrative records. For example, the Center for Sociological Research (CIS), which is a reference at the Spanish level, continues to use the sex variable (M and H) in its studies. Most studies carry out an analysis by sex (female and male) and there are still few authors who have considered the importance of an analysis by gender (García-Vega et al., 2005). In this sense, some authors consider that the inclusion of the independent variable “sex” in logistic regression models is not sufficient to explain the complexity of gender relations (Rohfl et al., 2000). Therefore, it is necessary to include the category gender.

As we have shown, the use of identification variables (sexual and gender) has evolved, and we find ourselves faced with new sociological realities to which the ways of questioning need to be adapted in order to capture all possible responses. Current research in the social sciences, gender-related issues, the increasing awareness of equality, and the growing literature make it clear that to categorize human beings into two options is outdated and ethically incorrect. Further, depending on each survey’s objective, analyses can be much more accurate if the demographic data are divided into more than two categories.

Nonetheless, given the broadness of the concept of GENDER, it can be difficult for researchers to develop appropriate questions and responses of gender options. What is clear is that when a question is drafted, it must be done in a respectful and inclusive manner.

There is now considerable consensus on the need to differentiate between the concepts of sex and gender. Gender, categorized as feminine or masculine, defines the stereotypes that a given culture at a given time considers to be associated with one or the other sex (Blanco, 2016). Thus, there are two relevant questionnaires that have been generated from the perspective of the double factor: the BSRI (Bem, 1974) and the EPAQ (Spence et al., 1979). These two scales continue to be the most widely used for the assessment of gender identity, although they are not free from criticism. Exempt from criticism, for example, the validity of the items at present or the disregard for the multidimensionality of the gender construct (Guerrero and Mirón, 2016). If we take into account sex-gender differentiation, we see that the most traditional concept that the various measurements from surveys and administrative records seek to approximate is that of biological sex. This concept, which refers to the body, indicates the sexual characteristics that people are born with, which are determined by their genital organs (internal and external), hormones, chromosomes and genes. The categories used to refer to this concept are: “Female” and “Male” (Instituto Nacional de Estadísticas, 2022).

Currently, the options that are most used in relation to the sex variable can be seen in the following examples:

**Table 1:** 

V. SEX	MALE	FEMALE	Other

The Basque Immigration Observatory (IKUSPEGI), for example, uses the option “Others” in its studies. Other studies such as one conducted by the Spanish Equality pf Ministry (2022) include the non-binary category.

**Table 2:** 

V. SEX	MALE	FEMALE	Non-binary

This is the simplest way of posing the question which aims to include a third response option, beyond the binary, without making the classification too long and that enables everyone to position themselves. However, using the term “other” or “non-binary” does not address or name new realities nor would individuals be given the visibility they deserve if they were included in a third, neutral or “not identified” group. Moreover, the “other” category seems to suggest a definition of otherness or peripheral of anything that does not fit the gender binary. For this reason, some surveys have started to include as another possible option the intersex category.

The measurement standards proposed by the Australian Bureau of National Statistics (ABS, 2021) and New Zealand (Stats NZ, 2021) have discouraged the measurement of intersex as a third sex. Instead, they propose that it should be addressed by means of an additional question to probe for the presence of variations in people’s sex characteristics. Thus, one of the basic principles of measurement is self-declaration, i.e., that it is the person himself/herself, whether or not he/she experiences the attribute, who provides the answer. This decision is based on the need for further research into the correct way to include the measurement of intersex, either as a third category in the “sex” variable or as an additional question (Instituto Nacional de Estadísticas, 2022).

**Table 3:** 

V. SEX	MALE	FEMALE	INTERSEX

Bayond sex, gender identity may or may not “match” with the biological sex or the sex assigned at birth. Internationally, the terms “transgender” and “cisgender” are used to classify people according to the relationship between sex and gender. In the case of direct respondent operations, this would be self-perceived gender (Instituto Nacional de Estadísticas, 2022).

In relation to the gender with which we identify, some response options are as follows:

**Table 4:** 

GENDER with which you identify	MALE	FEMALE	
GENDER with which you identify	MALE	FEMALE	OTHER
GENDER with which you identify	MALE	FEMALE	BIGENDER

Another classification is as follows (García et al., 2019):

**Table 5:** 

GENDER with which you identify	Male	Female	Androgynous	Undifferentiated

Bearing in mind that a person’s gender may or may not correspond with their sex assigned at birth, there are other, somewhat more developed options (Instituto Nacional de Estadísticas, 2022):

**Table 6:** 

GENDER with which you identify	Male	Female	Bigender	Transgender	Not sure	None	Prefer not to say

Some surveys include the variables listed above by incorporating a “time” element (at birth, or at the present time) (Instituto Nacional de Estadísticas, 2022).

**Table 7:** 

Biological sex you were born with		Female	Male	Intersex
**Gender assigned at birth:**		Female		Male
**According to your gender identity, you currently identify yourself as:**				
Female	Male	Transgender	Bigender	None

Where until recently the term sex was used, there is now a widespread tendency to replace it with gender and its derivatives, or for sex and gender to share space in scientific work, referring to two distinct domains. It is important to stress the need for a model capable of integrating both complex realities, that of sex and gender, as this approach can have important consequences in the field of research, in education, in the experience of women, men and ambiguous people, and in the clinical field (Fernández, 2010).

For those who do not feel comfortable or do not wish to be classified as a closed and specific option, there is also the option to leave the gender question open-ended so that each individual may express their own gender identity more freely. Another option is to use a continuum on a scale of 1 to 10. The difficulty then would be to quantitatively interpret the responses or establish classifications to capture patterns of behavior according to gender.

Finally, in relation to sexual orientation, some surveys capture this aspect as follows:

**Table 8:** 

According to your sexual orientation, you identify yourself as:					
Heterosexual	Homosexual	Bisexual			
Heterosexual	Gay	Lesbian	Bisexual	Not sure	Prefer not to say

In any case, what is clear is that the formulation of questions and answers should be adapted to the changes and new realities according to current times.

The Questionnaire of Attitudes towards Gender Equality (CAIG), elaborated by De Sola et al. (2003) includes the right to free choice in sexual orientation. According to the Instituto Nacional de Estadísticas (2022), in the context of the standard, the dimension that needs to be addressed is that of self-identification, in line with the need to make sexual diversities visible based on the self-perceived orientation of the informant at the time of the interview. Sexual orientation is also widely collected in the UK’s National LGTB Survey or in the EU-LGTBI Survey II.

## 4 The consequences of limiting sociodemographic variables: the case of the European values study

In addition to demonstrating how questions and answers relating to identities are formulated in surveys in general, it is interesting to present an example from a specific survey and examine how questions are posed and the limitations that these may impact the subsequent analyses, given they continue to follow a traditional format. To do this, we have chosen as a case study the European Values Study whose application in Spain is coordinated by our research team.

The European Values Study (EVS), that began in 1981 and has nearly 40 participating European countries, has not significantly evolved in the way sociodemographic identity questions are formulated. It only includes a question about the “sex of the respondent” with a binary response option: 1. Male 2. Female. It also includes “do not know,” “no response,” but only as a “spontaneous response,” that is, it is not asked by the interviewer and the reasons for the lack of response are not recorded.

The main justification for this approach given by surveys that have been conducted over decades, as is the case of the EVS, is the need to maintain the wording of the questions to guarantee comparability in longitudinal studies. This is a valid argument but does not justify the failure to include new question forms that allow for adaptation to theoretical and methodological developments and to a rapidly changing reality. One of the most relevant advances in critical social theory is the notion of intersectionality and the demand to apply an intersectional gender perspective in social research. This approach tends to be associated with a qualitative methodology; for example, when Sandra Harding defends Feminist Stand Theory, she notes three criteria that any feminist and inclusive investigation should meet (Silvestre et al., 2020). Firstly, it should provide new empirical resources based on the experiences of women and minorities, traditionally excluded from knowledge. Secondly, the investigation should bring new proposals to the field by positioning itself in favor of women, opposed to the traditional androcentric privilege, which implies a commitment for social transformation. Thirdly, it should provide a new object of study, situating the researcher on the same level as the object of study (subject/object of research relationship). The first and third criteria also challenge quantitative research. In the first case, the “experiences” of women and minorities are usually collected through qualitative techniques, however, quantitative research should not be excluded from this data collection and, to do this, it is necessary to understand the diversity of gender identities. In the third case, placing the subject and object of study on the same plane implies not only legitimizing the inclusion of diversity between those who research and theorize, it also entails legitimizing and including diversity in what is observed. To capture the diversity in what is observed, it is important to anticipate it in advance, as what is not sought is not found. This is where it becomes necessary to include a series of sociodemographic variables that include the different vectors of inequality that condition people’s lives, as those inequalities can be explanatory elements and significant predictors of the reality we are studying. In this sense, when the EVS asks about the degree of agreement of the statement “Homosexual couples are as good parents as other couples,” it cannot ignore that sexual orientation can be an explanatory variable in this question, as it also surely is when being asked to explain the degree of justification of “homosexuality.”

In this respect, the EVS, heir to the theoretical and methodological legacy of Ronald Inglehart, contains a set of questions that aims to study the change of values in society and the impact of modernity. Inglehart (1977) spoke of the “silent revolution” which associated socialization in the contexts of economic growth, with a change of values that entailed a shift from materialist values to post-materialist values. This process of modernization has also been linked to the process of secularization and, for example, in Spain, the relationship between moral permissiveness or openness and the process of secularization is very evident. Where religion is less important or less present there is greater moral permissiveness. In contrast, those who are more aware of religion and its dogmas tend to be governed by these and to be more morally strict, which is also linked to a conservative ideology (Silvestre et al., 2022). In summary, we know that changes in values are interwoven with religiosity, age and ideology but we cannot know if there is a relationship with gender identities, ethnicities or sexual orientation because this is not part of the question set.

## 5 Proposal of sociodemographic questions that include the categories of sex, gender and sexual orientation

It is a fact that certain questions may infringe on privacy and cause discomfort to respondents, but this is also the case in political questions such as vote recall. Ensuring anonymity and confidentiality of surveys, whilst meeting the required ethical criteria, is a way of promoting a climate of trust and honesty in respondents’ answers. In this regard, an approach to data collection that can guide us is the one proposed by the United Nations (2018) based on human rights to leave no one behind in the 2030 Agenda for sustainable development. This proposal upholds six principles: participation, data disaggregation, self-identification, transparency, privacy and accountability (United Nations, 2018, pp. 1–20).

1. Participation of relevant population groups in data collection exercises, including planning, data collection, dissemination and data analysis, that includes the assurance that the opinions of vulnerable and marginalized groups who are at risk of discrimination are represented.
2. Disaggregation of data allows users to compare population groups and to understand the situation of specific groups. On this point, it is noted that the collection of data to allow disaggregation may require alternative sampling and data collection approaches.
3. Self-identification: for the purposes of data collection, populations of interest should be self-defining. Individuals should have the option to disclose or withhold information about their personal characteristics. This point is key for the categories that we are dealing with and justifies the inclusion of open-ended questions that allow sexual and gender identity to be freely expressed (or not).
4. Transparency implies that data collectors should provide clear and accessible information about their operations, including the research design and data collection methodology. Further, data collected by State organizations should be accessible to the public.
5. Privacy is ensured when data is protected and kept private, ensuring confidentiality of individuals’ responses and personal information.
6. Accountability: data should be used to hold States and other actors to account on human rights issues. This refers to the second characteristic of Feminist Stand Theory which calls for a commitment to social transformation to achieve fairer and more egalitarian societies.

Not all surveys need to include an extensive set of sociodemographic questions; this should be determined by the object of the research, its objectives and stated hypotheses. However, if the study applies an intersectional gender approach, a broader set of questions may be beneficial to provide new empirical evidence. Finally, to achieve representativeness and reliability in every category undoubtedly makes sampling more complex and costly which is why the possibility of alternative sampling and data collection approaches are discussed.

Our proposal includes different ways of asking about sexual and gender identity to maintain comparability in longitudinal studies and official statistics. We recommend including the proposed set of questions in the same block with the following introductory text: “Below we ask you to answer a series of questions that aim to find out about your sexual and gender identity and your sexual orientation as we believe that these identities may shape and explain your ways of thinking and acting. Feel free to respond or not.

- Sex assigned at birth (female/male/“intersex”): this question aims to ensure longitudinal comparison with previous studies. In Spain, for real and effective equality of transgender people and to guarantee the rights of LGTBI people, the 4/2023 Law of 28 February delays sex assignment at birth to 12 years of age when there are doubts. In this case, the category “intersex” should also be included as there could be situations in which the male/female sex is not assigned.

- Gender identity:

○ Transgender
○ Bigender
○ Female
○ Male
○ Androgenous
○ Non
○ Do not know
○ Prefer not to say
○ Prefer to define myself as ………

- Do you identify as trans? (Instituto Nacional de Estadísticas, 2022)

○ Yes
○ No
○ Do not know
○ Prefer not to say

- Sexual orientation: we chose to open up the category “homosexual” to make visible lesbian women who often remain hidden behind this category.

○ Gay
○ Lesbian
○ Bisexual
○ Asexual
○ Heterosexual
○ Do not know
○ Prefer not to say
○ Prefer to define myself as ……….

## 6 Discussion and conclusion

As reflected above, the categorization of the population according to demographic data such as sex and/or gender is a very common practice, especially from a binary, cisgender, heteronormative approach. This dichotomous categorization, oversimplifying human diversity and its realities and reducing it to these two possibilities can have serious consequences and may compromise the scientific rigor of the entire research process, from sampling to the collection and analysis of information and, also, its results and conclusions. In addition, it renders non-normative groups and realities invisible.

It is for this reason that we consider it necessary to make a proposal such as the one presented here, applied to the European Values Survey or to any other survey.

We have formulated a proposal that aims to grasp different sexual and gender identities and diverse sexual orientations. We have combined closed and open-ended questions that favor self-assignment, as well as offering a non-response option. We believe that it is important to alternate the order of response options to avoid always asking about normative identities first. Our proposal allows comparability with previous studies and opens up the opportunity for in-depth intersectional analyses. One of the fears in introducing new ways of asking about identity is the loss of information for comparability with previous studies. Our proposal retains sex and introduces gender and thus allows comparability with previous studies. In other words, the potential use of this proposal is that it allows us to cross variables by sex and continue making diachronic comparisons, while at the same time introducing the analysis in terms of gender identity and sexual orientation, which is essential if we want to achieve an adequate understanding of the current social reality. Family dynamics, political experiences, or the phenomena of inequality and violence faced by these groups are just some examples of this. This would contribute to alleviating the scarcity of data on the lives of LGBTI people, which, as stated by the World Bank (2024), constitutes a fundamental obstacle to addressing stigma and exclusion based on sexual orientation and gender identity in different parts of the world.

It should also be noted that this proposal and the selection of the aforementioned categories is not based on statistical validity criteria, but on the epistemological issues discussed above. Statistical validity will be an *a posteriori* consequence of its application. To conclude, we believe that it is necessary to continue working on the current debate on this issue from a quantitative as well as a qualitative perspective as it is essential to continue measuring social reality in a way that is appropriate and adapted to the present times. A future line of research derived from our proposal could be the validation of its content by mixed techniques such as interviews or the validation by expert judgement. It should also be noted that statistical validity will be *a posteriori* consequence of its application.

## Author contributions

RR: Conceptualization, Supervision, Writing – original draft, Writing – review & editing. IA: Data curation, Formal analysis, Methodology, Writing – original draft, Writing – review & editing. MS: Conceptualization, Formal analysis, Funding acquisition, Resources, Supervision, Validation, Writing – original draft, Writing – review & editing.

## Footnotes

^1^As Platero notes, “our concepts of sex, sexuality and identity are constructed with many layers of history, geography, political relations, economic relations, etc.” (2013: 52).

^2^The term “gender” was coined by John Money (New Zealand doctor and psychologist) in 1955 in the context of his research on hermaphroditism, to designate modes of behaviour, expression and movement as well as play and conversation preferences that characterised masculine and feminine identities (Puleo, 2008, p. 15).

^3^Gender Studies introduced by the North American academy and the term “gender” were not without their critics who highlighted that this term blurred the activist nature and the social transformation of the “demonised” term “feminism” (Rodríguez Magda, 2015, pp. 25, 32).

^4^According to Laqueur (1994, cited in León, 2015), sex is a contextual construction – inseparable from the discursive medium – of the 18th century, when sexual differences between men and women were determined according to observable biological distinctions (previously it was thought that women had the same genitals as men inside their bodies).

^5^In her work “The Traffic in Women: Notes on the ‘Political Economy’ of Sex”, Rubin (1986) coined the concept sex/gender system which can be understood as “the set of arrangements by which a society transforms biological sexuality into products of human activity, and in which these transformed sexual needs are satisfied.” However, in her essay, “Thinking sex: Notes for a radical theory of the politics of sexuality,” although she admits that sex and gender are related, she considers it a mistake to have taken them for interchangeable cogs in the same process of social exclusion. She points out that gender, as a category of analysis, is insufficient to explain the sexual oppression that arises as a result of individuals’ sexual orientation (Rubin, 1989). She recounts that in the US from the late 1940s to 1960, alongside communists, homosexuals were the “object of purges and witch hunts throughout the country” (1989: 118–119). The questionable spectre of the sex offender that was applied to rapists and child molesters also functioned as a code for homosexuals, in this way blurring the distinction between sexual assault and illegal, although consensual, acts such as sodomy (Rubin, 1989).

^6^For Rubin (1989) this is reflected in public policies and in cultural representations.

^7^Nevertheless, part of lesbian theory proposes an essentialist vision of lesbians (Álvarez, 2001, p. 275).

^8^For this author, heterosexual society is based on the necessity of the other/different, who is no other than the dominated. To construct and control difference is in itself an act of power, essentially a normative act. The concept of sex differences constitutes women as others/different; men are not different, in the same way that whites are not different, nor the masters (in contrast to blacks and slaves). “For us, this means there cannot any longer be women and men, and that as classes and categories of thought or language they have to disappear, politically, economically, ideologically. If we, as lesbians and gay men, continue to speak of ourselves and to conceive of ourselves as women and as men, we are instrumental in maintaining heterosexuality” (Wittig, 2009, p. 140).

^9^Some of the objections to Butler’s theory have questioned the voluntarism or the radical deconstruction of the body that they confronts in their work “Bodies that Matter,” and whilst this theory focuses on the issue of recognition rather than redistribution, what is lacking is the connection between the themes that they address and the current economic reality of capitalist societies (Guerra and Fernández, 2022).

^10^As Tubert (2003) questions, referring to sex at the biological level and gender as a cultural fact reproduces the nature-culture dichotomy.

^11^We should also mention Azpiazu (2017) and his work “Masculinidades y feminismo”.

^12^For Ana de Miguel (2014) only from a “we,” which allows us to theorise on “what unites women,” is it possible to develop the diversity and intersectionality of feminism and understand the ways in which oppression manifests in women’s lives (191). She considers that the proliferation of identities on which queer theory focuses weakens this political subject and that its claims do not represent the majority of women, as shown by its lack of meaning for “the millions of women in the world who cannot control men’s access to their bodies” (De Miguel, 2014, p. 199).

## References

[ref9001] ABS. (2021). Standard for Sex, Gender, Variations of Sex Characteristics and Sexual Orientation Variables. Recuperado el 1 de Diciembre de 2021, de Australian Bureau of Statistics. Available at: https://www.abs.gov.au/statistics/standards/standard-sex-gender-variations-sex-characteristics-and-sexual-orientation-variables/latest-release.

[ref1] Álvarez S. (2001). “Diferencia y teoría feminista [difference and feminist theory] in Feminismos” in Debates teóricos contemporáneos [feminisms. Contemporary theoretical debates]. eds. Beltrán E. Maquieira V. (Madrid: Alianza), 243–286.

[ref2] Andalzúa G. (1987). Borderlands/La Frontera: The new mestiza. San Francisco: Spinster/Aunt Lute.

[ref3] Azpiazu J. (2017). Masculinidades y femenismo [Masculinities and feminism]. Barcelona: Virus.

[ref4] Bagiotto V. (2019). Butler: Cuerpos que importan o sobre la pregunta por la materialidad del cuerpo [Butler: bodies that matter or on the question of the materiality of the body]. Reflexiones Marginales, 54. Available at: https://revista.reflexionesmarginales.com/butler-cuerpos-que-importan-o-sobre-la-pregunta-por-la-materialidad-del-cuerpo/

[ref9002] Bem S. L. (1974). The measurement of psychological androgyny, J. Consult. Clin. Psychol., 42, 155–162. 4823550

[ref9003] Blanco M San Segundo R. (2016). Investigación joven con perspectiva de género. Instituto de estudios de género. Universidad Carlos III de Madrid. Madrid.

[ref5] Butler J. (1990). Gender trouble: Feminism and the subversion of identity. New York: Routledge.

[ref6] Butler J. (2022). Cuerpos que importan. Sobre los límites discursivos del “sexo” [Bodies that matter: On the discursive limits of “sex”]. Barcelona: Editorial Planeta.

[ref7] Butler J. Fraser N. (2000). ¿Redistribución o reconocimiento? Un debate entre marxismo y feminismo [Redistribution or Recognition? A Political-Philisophical Exchange]. Madrid: Editorial Traficantes de Sueños.

[ref8] Carbado D. W. Crenshaw K. W. Mays V. M. Tomlinson B. (2013). Intersectionality. Mapping the movements of a theory. Du bois review social science research on race 10, 303–312. doi: 10.1017/S1742058X13000349, PMID: 25285150 PMC4181947

[ref9] Cobo R. (1995). Género [gender] in Diez palabras clave sobre mujer [ten keywords about women]. Estella: Verbo Divino, 55–83.

[ref10] Coll-Planas G. (2010). La voluntad y el deseo. La construcción social del género y la sexualidad: el caso de lesbianas, gays y trans [Will and desire. The social construction of gender and sexuality: the case of lesbians, gays and trans]. Madrid: Editorial Egales.

[ref11] Combahee River Collective (1981). A black feminist statement in this bridge called my Back: Writings by radical women of color. New York: Kitchen Table, Women of Color Press, 210–218.

[ref12] Connell R. W. (1995). Masculinities. Cambridge: Polity Press.

[ref13] Crenshaw K. W. (1989). Demarginalizing the intersection of race and sex: a black feminist critique of antidiscrimination doctrine, feminist theory and antiracist politics. Univ. Chic. Leg. Forum. 1989, 139–168.

[ref14] Davis A. Y. (1983). Women, race and class. New York: Vintage Books.

[ref15] De Beauvoir S. (1972). The second sex. Harmondswick, UK: Penguin.

[ref16] De Miguel A. (2014). La dialéctica de la Teoría Feminista: Lo que nos Une, lo que nos separa, lo que nos hace avanzar [the dialectics of feminist theory: what unites us, what separates us, what moves us forward]. Daimon. Revista Internacional Filosofía 63, 191–204. doi: 10.6018/daimon/199711

[ref17] De Sola A. Martínez I. Meliá J. L. (2003). El cuestionario de actitudes hacia la igualdad de géneros (CAIG): elaboración y estudio psicométrico. Anuario Psicol. 34, 101–123.

[ref18] Fernández J. (2010). El sexo y el género: dos dominios científicos diferentes que debieran ser clarificados. Psicothema 22, 256–262. Available at: http://www.psicothema.com 20423630

[ref19] Galcerán M. (2000). “¿Qué se reconoce en las políticas de reconocimiento? Una introducción al debate entre Nancy Fraser y Judith Butler [what is recognized in recognition politics? An introduction to the debate between Nancy Fraser and Judith Butler]” in in ¿Redistribución o reconocimiento? Un debate entre marxismo y feminism [Redistribution or Recognition? A Political-Philisophical Exchange]. eds. Butler J. Fraser N. (Madrid: Editorial Traficantes de Sueños), 7–22.

[ref20] Gandarias I. (2017). ¿un neologismo a la moda?: Repensar la interseccionalidad Como herramienta Para la articulación política feminista [a trending neologism? Rethinking intersectionality as a tool for feminist political articulation]. Investig. Feministas 8, 73–93. doi: 10.5209/INFE.54498

[ref21] García F. E. Alzugaray C. Ornella C. Espinoza B. Salgado G. Garabito S. (2019). ¿Masculino, femenino, andrógino o indiferenciado? Relación entre el rol sexual, la afectividad y la inteligencia emocional en personas adultas [masculine, feminine, androgynous or undifferentiated? The relation between sexual role, affectivity and emotional intelligence in adults]. Psychología 13, 55–65. doi: 10.21500/19002386.4001

[ref22] García-Vega E. Fernández P. Rico R. A. (2005). Género y sexo como variables moduladoras del comportamiento sexual en jóvenes universitarios. Psicothema 17, 49–56. Available at: http://www.psicothema.com

[ref23] Giddens A. (2002). Sociología [Sociology]. Madrid: Alianza Editorial.

[ref24] Guerra V. D. Fernández S. Y. (2022). Más allá de Judith Butler: reflexiones sobre “El género en disputa. Feminismo y subversión de la identidad” [beyond Judith Butler: reflections on gender trouble. Feminism and the subversion of identity]. La Manzana Discordia. 16, 1–30. doi: 10.25100/lamanzanadeladiscordia.v16i2.11510

[ref25] Guerrero Y. Mirón M (2016). Análisis y evaluación de las características, dimensiones y correlatos de la identidad de género. In Blanco M. Segundo R San, (Eds). Investigación joven con perspectiva de género. Instituto de estudios de género. Madrid: Universidad Carlos III de Madrid. 177–198.

[ref26] Hill Collins P. H. (1990). Black feminist thought. Knowledge, consciousness and the politics of empowerment. Boston: Unwin Hyman.

[ref27] Hill Collins P. H. Bilge S. (2016). Intersectionality. United States: Polity Press.

[ref28] Hooks B. (1981). Ain’t I a woman. Black women and feminism. Boston: South End Press.

[ref29] Inglehart R. (1977). The silent revolution: Changing values and political styles among Western publics. Princeton: Princeton University Press.

[ref30] Instituto Nacional de Estadísticas. (2022). Estandarización de preguntas para la medición de género, sexo y orientación sexual (SGOS), dirigido a hogares y censos de población [Question standardization for the measurement of gender, sex and sexual orientation (SGOS) for households and population censuses]. Instituto Nacional de Estadísticas de Chile. Availabe at: https://www.ine.gob.cl/docs/default-source/buenas-practicas/directrices-metodologicas/estandares/documentos/estandarizaci%C3%B3n-de-preguntas-

[ref9005] Jackson S. Scott S. (2010). Theorizing sexuality. Glasgow: Open University Press.

[ref31] La Barbera M. C. (2016). Interseccionalidad, un “concepto viajero”: orígenes, desarrollo e implementación en la Unión Europea. [intersectionality, a “traveling concept”: origins, development and implementation in the European Union]. Interdisciplina 4, 105–122. doi: 10.22201/ceiich.24485705e.2016.8.54971

[ref32] Lagarde y de los Ríos M. (2000). Claves feministas para la autoestima de las mujeres [Key feminist concepts for women’s self-esteem]. Madrid: Horas y Horas.

[ref9008] Laqueur T. (1994). La construcción del sexo. Cuerpo y género desde los griegos hasta Freud [The construction of sex. Body and gender from the Greeks to Freud]. Madrid: Cátedra.

[ref33] León M. E. (2015). Breve historia de los conceptos de sexo y género [brief history of sex and gender]. Revista Filos. Universid. Costa Rica 138, 39–47.

[ref34] López Sáez M. A. García-Dauder D. (2020). Los test de masculinidad/feminidad como tecnologías psicológicas de control de género [Mascuinity/femeninity tests as psychological technologies in gender control]. Athenea Digital 20, 2521–2530. doi: 10.5565/rev/athenea.2521

[ref35] Maquieira V. (2001). “Género, diferencia y desigualdad [gender, difference and inequality] in Feminismos” in Debates teóricos contemporáneos [feminisms. Contemporary theoretical debates]. eds. Beltrán E. Maquieira V. (Madrid: Alianza), 127–184.

[ref9009] McCall L. (2005). The Complexity of Intersectionality, Signs: Journal of Women in Culture and Society, 30, 1771–1800.

[ref36] Mill J. S. (2001). “La sujeción de las mujeres [the subjection of women]” in Ensayos sobre la igualdad sexual [Essays on sexual equality]. eds. Mill J. S. Taylor H. (Madrid: Cátedra), 149–258.

[ref37] Millet K. (2000). Sexual politics. Champaign, IL: University of Illinois.

[ref38] Ministerio de Igualdad (2022). Estudio sobre las necesidades de las personas no binarias en España. Madrid: Dirección General de Diversidad y Derechos LGTBI.

[ref39] Momoitio (2019). “Lesbianismo y otras proezas [Lesbianism and other exploits] in Feminismos” in Miradas desde la diversidad [Feminisms. Perspectives from diversity] Ed. Fernández J. (Madrid: Ediciones Oberon), 29–41.

[ref40] Moraga C. Andalzúa G. (1981). This bridge called my Back: Writings by radical of color. New York: Kitchen Table, Women of Color Press.

[ref9004] Nash J. (2010). On difficulty: Intersectionality as a feminist labor. The scholar and feminist online, 8, 1–10.

[ref41] Oliva A. (2005). Debates sobre el género [Gender debates] in Teoría feminista: de la ilustración a la globalización. De los debates sobre el género al multiculturalismo [Feminist Theory: from illustration to globalization. Debates from gender to multiculturalism]. Madrid: Minerva, 13–60.

[ref42] Platero R. L. (2012). La interseccionalidad como herramienta de estudio de la sexualidad [Intersectionality as a tool to study sexuality] in Intersecciones: cuerpos y sexualidades en la encrucijada [Intersections: bodies and sexualites at the crossroad]. Barcelona: Edicions Bellaterra, 15–72.

[ref43] Platero R. L. (2013). Marañas con distintos acentos. Género y sexualidad en la perspectiva interseccional [tangled messes with different accents. An intersectional approach to gender and sexuality]. Encrucijadas. Revista de Ciencias Sociales 5, 44–52.

[ref44] Poulain de la Barre F. (1984). De l’égalité des deux sexes [of equality of the two sexes]. París: Fayard.

[ref45] Puleo A. (2008). El reto de la igualdad de género. Nuevas perspectivas en ética y filosofía política [the challenge of gender equality. New perspectives on ethics and political philosophy]. Madrid: Biblioteca Nueva.

[ref46] Rich A. (1999). La heterosexualidad obligatoria y la existencia lesbiana [Compulsory heterosexuality and lesbian existence] in Sexualidades, género y roles sexuales [Sexuality, gender and sexual roles]. Buenos Aires: Fondo de Cultura Económica, 159–212.

[ref47] Rivera Garretas M. M. (1994). Nombrar el mundo en femenino: Pensamiento de las mujeres y teoría feminista [naming the world feminine. Women’s thoughts and feminist theory]. Barcelona: Icaria.

[ref48] Rodríguez Magda R. M. (2015). Sin género de dudas [without any doubt. Successes and challenges of feminism today]. Madrid: Biblioteca Nueva.

[ref49] Rohfl I. Borrell C. Anitua C. (2000). La importancia de la perspectiva de género en las encuestas de salud. Gac. Sanit. 14, 146–155. doi: 10.1016/S0213-9111(00)71448-8 10804105

[ref50] Royo R. (2012). Maternidad, paternidad y conciliación en la CAE. ¿Es el trabajo familiar un trabajo de mujeres? [maternity, paternity and conciliation in the CAE. Is family work woman’s work?]. Bilbao: Universidad de Deusto.

[ref51] Rubin G. (1986). El tráfico de mujeres. Notas sobre la “economía política” del sexo [the traffic in women: notes on the “political economy” of sex]. Nueva Antropol 30, 95–145.

[ref52] Rubin G. (1989). Reflexionando sobre el sexo: Notas Para una teoría radical de la sexualidad [thinking sex: Notes for a radical theory of the politics of sexuality] in placer y peligro: Explorando la sexualidad femenina [pleasure and danger: Exploring female sexuality]. Madrid: Revolución, 113–190.

[ref53] Severs E. Celis K. Erzeel S. (2017). Poder, privilegio y desventaja. Teoría interseccional y representación política [Power, privilege and disadvantage. Intersectional theory and political representation]. Investigaciones Feministas 8, 41–51. doi: 10.5209/INFE.56400

[ref54] Silvestre M. Bartolomé E. Elzo J. (2022). Modernization and secularization in Spain: Evidence from values surveys in Reflections on European values: Honouring Loek Halman’s contribution to the European values study. Tilburg: Open Press TiU, 135–149.

[ref55] Silvestre M. López M. Royo R. (2020). The application of feminist standpoint theory in social research. Investigaciones Feministas 11, 307–318. doi: 10.5209/infe.66034

[ref56] Silvestre M. López M. Royo R. (2021). New feminist studies in audiovisual industries|the fourth wave in audiovisual content: a true achievement of feminism? Int. J. Commun. 15, 416–438.

[ref9010] Spanish Equality pf Ministry (2022). Estudio sobre las necesidades de las personas no binarias en España. [Study on the needs of non-binary people in Spain], Madrid: Dirección General de Diversidad y Derechos LGTBI.

[ref9006] Spence J. T. Hemreilch R. M. Holahand C. K. (1979). Negative and positive components of pshycological masculinity and feminity, and the relationship to shelp-reports of neurotic and acting out behaviours. J. Pers. Soc. Psychol., 37, 1673–1682. 512834

[ref57] Stang F. (2019). La invisibilidad estadística de la diversidad sexual y de género en los censos latinoamericanos: experiencias y algunas recomendaciones frente a la ronda censal 2020 in CEPAL, Aspectos conceptuales de los censos de población y vivienda: desafíos para la definición de contenidos incluyentes en la ronda 2020 [The invisible statistics of sex and gender diversity in Latin American censuses: experiences and recommendations from the 2020 census round]. Santiago de Chile: Serie Seminarios y Conferencias.

[ref9007] Stats N. Z. (2021). Statistical standar for gender, sex, and variations of sex characteristics. Wellington, New Zealand: Stats NZ Tatauranga Aotearoa.

[ref58] Suarez M. (2019). Discursos de sexualidad y poder. Continuidades y rupturas del orden sexual heteropatriarcal [discourses of sexuality and power. Continuities and ruptures of the heteropatriarchal sexual order] Unpublished doctoral thesis. Bilbao: Universidad de Deusto.

[ref59] Tubert S. (2003). Del sexo al género. Los equívocos de un concepto [from sex to gender. The misunderstandings of a concept]. Madrid: Cátedra.

[ref60] United Nations. (2018). A Human Rights-Based Approach to Data. Leaving No One Behind in the 2030 Agenda for Sustainable Development. Available at: https://www.ohchr.org/sites/default/files/Documents/Issues/HRIndicators/GuidanceNoteonApproachtoData.pdf

[ref61] Velasco S. (2009). Sexo, género y salud [sex, gender and health]. Madrid: Minerva.

[ref62] Wittig M. (2009). El pensamiento heterosexual (1978–1980) [heterosexual thought (1978–1980)] in Manifiestos gays, lesbianos y queer. Testimonios de una lucha (1969–1994) [gay, lesbian and queer manifestos. Testimonios of a struggle (1969–1994)] Icaria Editorial, 133–143.

[ref63] Wollstonecraft M. (2005). Vindicación de los derechos de la mujer [a vindication of the rights of women]. Madrid: Itsmo.

[ref64] World Bank (2024). Orientación sexual e identidad de género [Sexual orientation and gender identity], Available at: https://www.bancomundial.org/es/topic/socialdevelopment/brief/sexual-orientation-and-gender-identity

